# Methanol-specific methyltransferase isozymes have large carbon kinetic isotope effects that impact methane isotopic signatures

**DOI:** 10.1128/aem.00664-26

**Published:** 2026-07-21

**Authors:** Jonathan Gropp, Markus Bill, Daniel A. Stolper, Dipti D. Nayak

**Affiliations:** 1 Department of Earth and Planetary Science, University of California, Berkeley, California, USA; 2 Department of Molecular and Cell Biology, University of California, Berkeley, California, USA; 3 Energy Geosciences Division, Lawrence Berkeley National Laboratory 1666, Berkeley, California, USA; 4 Department of Plant and Microbial Biology, University of California, Berkeley, California, USA; Georgia Institute of Technology, Atlanta, Georgia, USA

**Keywords:** Methanosarcina, kinetic isotope effect, methyltransferases, methylotrophic methanogenesis, methanogen

## Abstract

The stable carbon and hydrogen isotopic composition of methane is widely used to determine its sources. Methanogenic growth on methanol generates methane with significantly lower ^13^C/^12^C ratios relative to other substrates, which is used as a marker for this metabolism in environmental samples. The biochemical basis for the large carbon isotope effect associated with methanol growth is currently unknown. Here, we measure the change in the carbon and hydrogen stable isotopic compositions of the methane produced by *Methanosarcina acetivorans* during growth on methanol. We couple these results with an inverse modeling approach to calculate the kinetic isotopic effects (KIEs) of the rate-limiting step, catalyzed by the methanol-specific methyltransferase complex (MTA). Through this process, we estimate the carbon KIE of MTA (^13^*ε*_MTA_) to be −66‰ and the hydrogen KIE of MTA (^2^*ε*_MTA_) to be −56‰. Our results indicate that the ^13^ε_MTA_ contributes substantially to the large carbon isotope effect observed on methanol. We also show that mutant strains that express only a single copy of the MTA complex (MtaC1B1A1, MtaC2B2A1, or MtaC3B3A1) have ^13^*ε*_MTA_ and ^2^*ε*_MTA_ that are indistinguishable from the wild-type strain. Finally, we propose that methanol activation by MTA will remain rate-limiting, even at low environmental methanol concentrations, and the large ^13^*ε*_MTA_ would be expressed *in situ* as well.

**IMPORTANCE:**

Mapping the biological sources of methane is vital for accurately modeling global emissions of this potent greenhouse gas. Methanogenic growth on methanol produces methane with a uniquely depleted carbon isotope signature, which is commonly used as an environmental indicator of this metabolism. The biochemical mechanisms driving this isotopic signature have remained unclear. In this study, we identify the methanol-specific methyltransferase (MTA) complex as the primary driver of these large carbon isotope effects in the model methanogen, *Methanosarcina acetivorans*. By utilizing *M. acetivorans* mutants that express a single MTA complex, we demonstrate that these effects are consistent across MTA isozymes that are expressed during different growth phases. Our results suggest these isotopic signatures are intrinsic to the enzyme complex and persist in natural environments. These findings provide a critical biochemical foundation for using stable isotopes to track methane production from methanol in diverse natural ecosystems.

## INTRODUCTION

Stable isotopic ratios of carbon (^13^C/^12^C) and hydrogen (^2^H/^1^H) are commonly used to trace biological and geological sources of methane in the environment (e.g., references 1–3). Methanogenic archaea (methanogens) are the primary source of biogenic methane, and the carbon and hydrogen isotopic composition of the methane that they produce can vary substantially (1). This variability stems, in part, from the enzyme-specific kinetic isotope effects (KIEs) associated with catabolic enzymes involved in methanogenesis, where KIEs are defined as the ratio of the rate constants for a given reaction for the heavy vs light isotope (k*_h_* and k*_l_*, respectively, where KIE = k*_h_*/k*_l_*). In addition to enzymatic KIEs, the isotopic composition of microbial methane also depends on pathway topology, that is, if there are any branch points where carbon is diverted to an end product other than methane, and the degree of reversibility for a given enzyme, which is set by the free energy change associated with the reaction catalyzed (∆G) (4–7). The extent to which each of these parameters influences the isotopic composition of methane depends on a variety of factors such as substrate availability and its initial isotopic composition, as well as temperature, pH, and other environmental conditions (6, 8–10). Under substrate-replete conditions, such as those typically used for laboratory batch cultures, enzymatic KIEs and pathway topology are generally understood to be the main determinants of the isotopic composition of methane relative to the substrate (7).

The combined effects of KIEs and pathway topology on the isotopic composition of methane can be quantified by the isotopic difference between the substrate and methane, which is reported using the ε notation as shown in equation 1:


(1) $${}^{13}\varepsilon_{CH4\text{-}substrate}={\left(\frac{\left[{}^{13}C\right]}{\left[{}^{12}C\right]}\right)}_{CH4}/{\left(\frac{\left[{}^{13}C\right]}{\left[{}^{12}C\right]}\right)}_{substrate}-1$$

**Fig 1:**
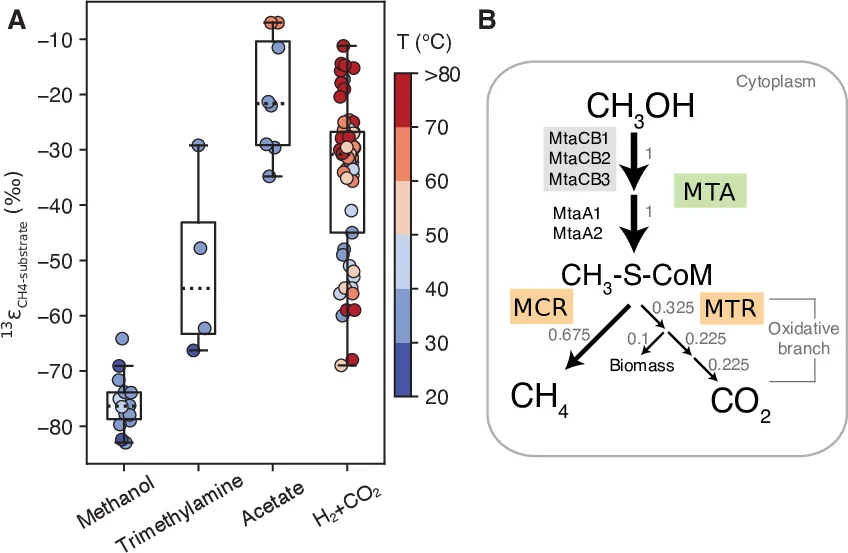
Isotopic effects of microbial methane production. (**A**) Measured values of ^13^*ε*_CH4-substrate_ for different methanogenic carbon sources in axenic laboratory cultures. The box plots represent the 25th, 50th, and 75th percentiles of the data. The colors represent the growth temperature (Data from references 4, 6, 8, 12, 14–26, 36, 37; see full list in Table S1). We do not provide data for hydrogen isotopes as the hydrogen isotopic compositions of methane generated from methylated compounds and acetate are controlled by the *δ*D of both the organic substrate and the water. Hence, the hydrogen isotopes can vary depending on the *δ*D of the water used for growth. (**B**) Our simplified metabolic pathway for methylotrophic methanogenesis on methanol in *Methanosarcina acetivorans*. The methyl group from methanol is activated by a methanol-specific MTA complex to methyl-coenzyme M (CH_3_-S-CoM). The complex includes MtaCB, which has three isoforms in *M. acetivorans*, and MtaA, which has two isoforms. The CH_3_-S-CoM is either reduced to CH_4_ or oxidized to CO_2_ in a 3:1 ratio (gray numbers next to the arrows indicate the relative flux through the reaction). The more detailed metabolic pathway used for data fitting is given in Fig. S1. MTR—N^5^-methyl-tetrahydromethanopterin:coenzyme M methyltransferase; MCR—methyl-coenzyme M reductase.

During laboratory cultivation of methanogens, which typically occurs in batch culture at high substrate concentrations, methanol growth produces the largest known values of ^13^*ε*_CH4-substrate_ (between ≈−65‰ and ≈−85‰) (4, 11–13) compared to ^13^*ε*_CH4-substrate_ of ≈−35‰ for acetate, ≈−65‰ for trimethylamine, and ≈−70‰ for H_2_/CO_2_ (Fig. 1A) (4, 6, 8, 12, 14–26). Methanogenic growth on methanol is known to be an important source of methane in environments such as salt marshes, marine sediments, and mangrove forests, and is likely even more widespread (e.g., references 27–35). The distinct ^13^*ε*_CH4-substrate_ values observed for this pathway could be critical to assess its contribution to global methane budgets. However, the molecular and biochemical rationale for the large carbon isotopic offset on methanol is poorly understood, limiting its utility as a tracer. Resolving this biochemical knowledge gap is the focus of this study.

During methanol growth, the substrate likely diffuses across the cell envelope and enters the cytosol where a methanol-specific methyltransferase (MtaB) catalyzes a methyl-transfer reaction to a corrinoid-containing protein, MtaC (Fig. 1B). There are three copies of the MtaCB complex in the model organism used for this study, *Methanosarcina acetivorans,* which are encoded by *mtaC1B1*, *mtaC2B2*, and *mtaC3B3* (Fig. 1B). The methyl-group bound to MtaC is subsequently transferred to coenzyme M (HS-CoM) to form methyl–coenzyme M (CH_3_-S-CoM) by a second methyltransferase (MtaA) (Fig. 1B). There are two copies of MtaA in *M. acetivorans,* which are encoded by *mtaA1* and *mtaA2* (Fig. 1B). Since the two methyltransferase reactions act in concert, here we refer to the combination of MtaCB and MtaA as the MTA complex. The methyl groups in CH_3_-S-CoM produce methane and carbon dioxide in a 3:1 ratio to maintain redox balance (38). The reduction of CH_3_-S-CoM to methane is catalyzed by MCR, and the stepwise oxidation of the methyl group in CH_3_-S-CoM to CO_2_ is catalyzed by a series of six enzymes that we collectively term the “oxidative branch” (Fig. 1B; Fig. S1).

Because MTA is the only enzyme complex in the methanogenic pathway that is unique to growth on methanol, Krzycki et al. (4) proposed that the KIE of MTA underlies the large ^13^*ε*_CH4-substrate_ values observed on methanol. Penger et al. (13) proposed that the disproportionation of CH_3_-S-CoM to CH_4_ and CO_2_ might additionally contribute to the large ^13^*ε*_CH4-substrate_ values associated with methanogenesis on methanol. Importantly, the branched pathway topology for growth on methanol is a common feature of methylotrophic methanogenesis (methanogenic growth on methylated substrates) on other substrates like trimethylamine. Growth on these substrates does not produce the large ^13^*ε*_CH4-substrate_ values seen for growth on methanol (Fig. 1A). As such, we consider it unlikely that the branched topology of methylotrophic methanogenesis is the sole origin of the large ^13^*ε*_CH4-substrate_ observed for growth on methanol (4). As articulated by Krzycki et al. (4), the more parsimonious explanation is that MTA is the main isotopically discriminating step in the pathway under typical laboratory growth conditions. If MTA controls ^13^*ε*_CH4-substrate_, then the isotopic composition of methane vs methanol will be set by a composite of the three MtaCB isozyme-specific isotopic effects. These three isozymes are actively expressed during growth on methanol, and their relative abundances change during different growth phases (39, 40). Thus, the isotopic effects expressed might change depending on the dominant MtaCB. Constraints on the isotopic effect of each isozyme would thus be necessary to use carbon isotopes as reliable tracers of methanogenesis on methanol.

To this end, here we measure KIEs for MTA in *M. acetivorans* using a combination of experimental measurements and an inverse modeling approach with the assumption that, under substrate-replete conditions, there is a single rate-limiting step for methanol-mediated methanogenesis (41). We find that the carbon KIEs of the MTA complex are relatively large (≈−65‰) and similar across the isozymes. These findings support the hypothesis that the large carbon KIEs of MTA dominate the carbon isotope effects observed during methanogenic growth on methanol.

## RESULTS

### Growth parameters and isotopic compositions of methane from *M. acetivorans* strains that express single methyltransferase isozymes

**Fig 2:**
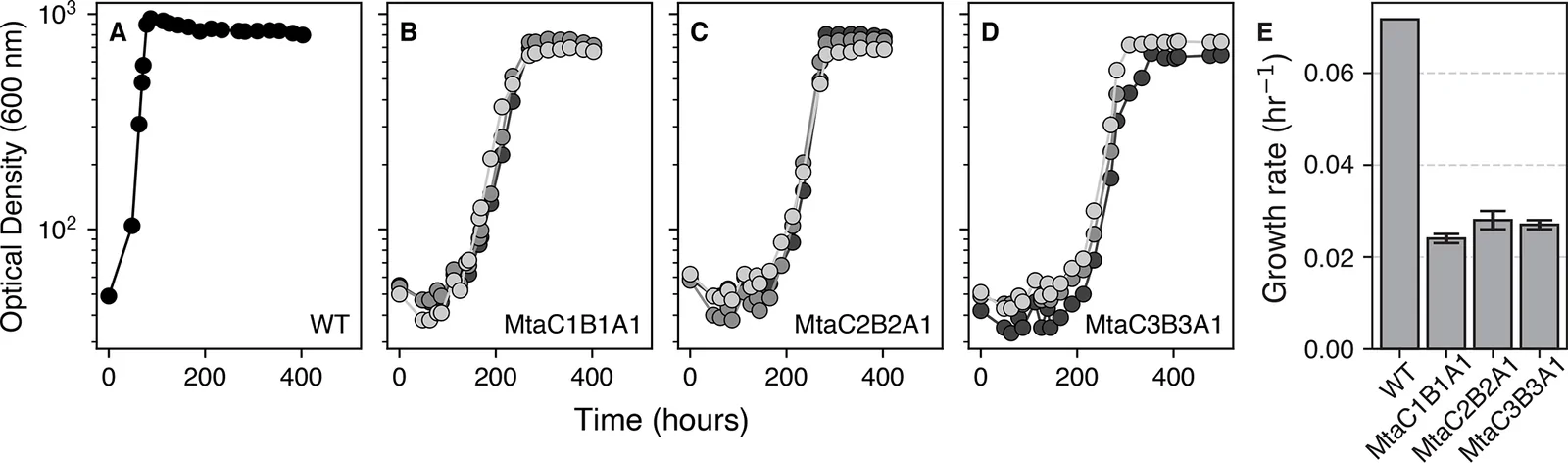
Growth and methane production data for *M. acetivorans* strains expressing different methyltransferase isozymes. (**A–D**) Representative growth curves where each color represents a single biological replicate. Cells were grown in HS minimal medium with 125 mM methanol at 37°C. All growth curves are provided in Fig. S2. (**E**) Growth rates of WWM1 (WT), WWM176 (expressing only MtaC1B1A1), WWM170 (expressing only MtaC2B2A1), and WWM184 (expressing only MtaC3B3A1), where error bars represent 1 standard error of the mean (SEM, *n* = 3) (see Table 1 for a list of strains used here).

All growth experiments were conducted at 37°C in hermetically sealed Balch tubes containing 10 mL minimal high-salt (HS) medium supplemented with 125 mM methanol as the sole carbon and energy substrate. We designate WWM1 (42) as the “wild-type” (WT) strain of *M. acetivorans* as it is the parent for mutant strains expressing only one of the three *mtaCB* operons along with *mtaA1* under a minimal *mcrB* promoter (WWM170, WWM176, and WWM184; reference 43, genotypes listed in Table 1). All three mutant strains had ~3-fold slower growth rates and ~5-fold longer lag times relative to the WT, which is consistent with a prior study that generated and characterized these mutants (Fig. 2A through E; Tables S2 and S3) (43).

**TABLE 1:** *Methanosarcina acetivorans* strains used in this study

Strain	Genotype	Ref.
WWM1	Δ*hpt*	(42)
WWM170	Δ*mtaC1B1* Δ*mtaC2B2* Δ*mtaC3B3* Δ*mtaA1* Δ*mtaA2* Δ*hpt*::P*mcrB-tetR*-𝜙C31*int-attP*::P*mcrB*(*tetO1*)-*mtaC2B2A1*	(43)
WWM176	Δ*mtaC1B1* Δ*mtaC2B2* Δ*mtaC3B3* Δ*mtaA1* Δ*mtaA2* Δ*hpt*::P*mcrB-tetR*-𝜙C31 *int-attP*::P*mcrB*(*tetO1*)-*mtaC1B1A1*	(43)
WWM184	Δ*mtaC1B1* Δ*mtaC2B2* Δ*mtaC3B3* Δ*mtaA1* Δ*mtaA2* Δ*hpt*::P*mcrB-tetR*-𝜙C31 *int-attP*::P*mcrB*(*tetO1*)-*mtaC3B3A1*	(43)

We tracked the carbon isotopic composition of the methane produced over the course of a growth cycle using isotope-ratio mass spectrometry (IRMS) and report it using the delta (δ) notation where:


(2) $$\delta^{13}C_{CH4}={\left(\frac{\left[{}^{13}C\right]}{\left[{}^{12}C\right]}\right)}_{CH4}/{\left(\frac{\left[{}^{13}C\right]}{\left[{}^{12}C\right]}\right)}_{VPDB}-1$$

**Fig 3:**
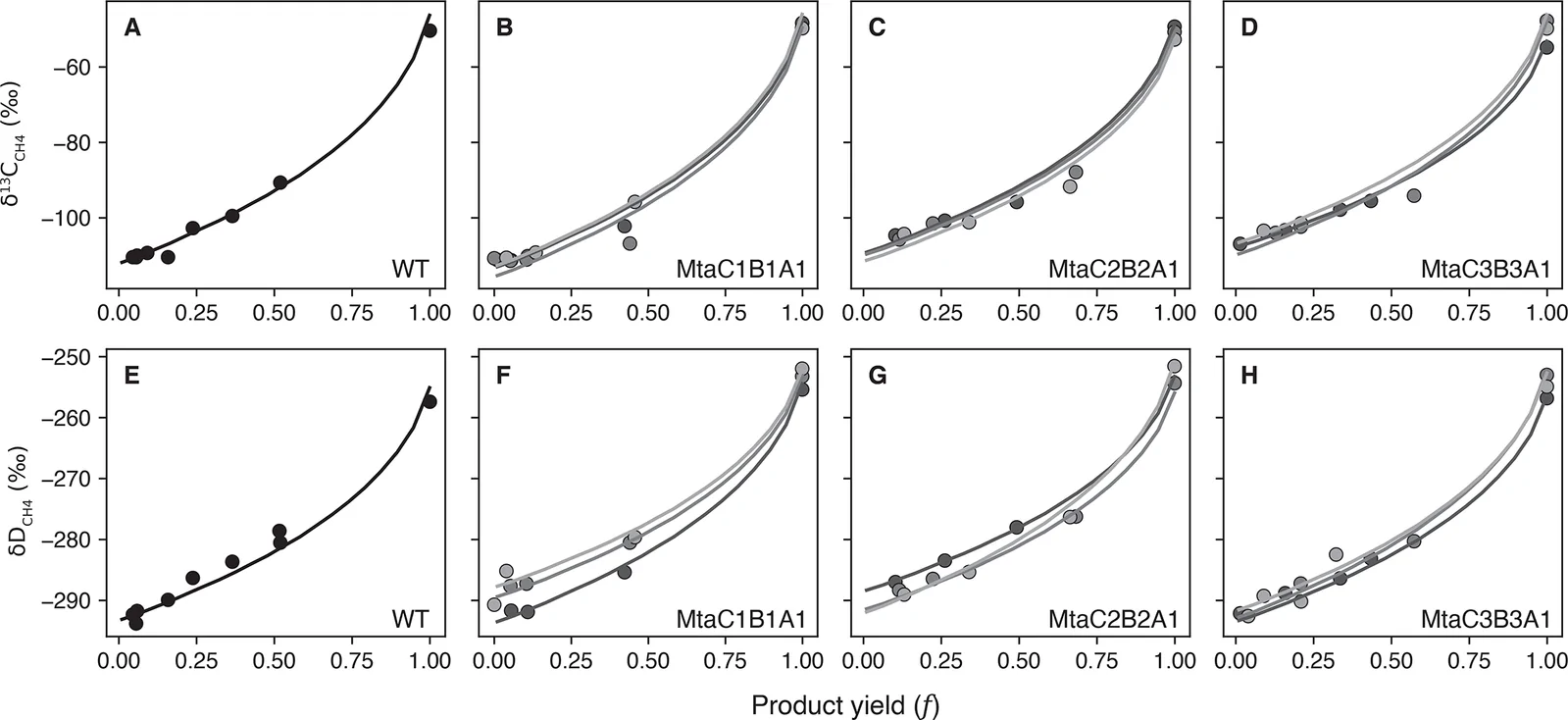
Isotopic composition of methane in the wild type and mutants that express single methanol-dependent methyltransferase isozymes. Measured (circles) and model fit (lines) (**A–D**) *δ*^13^C_CH4_ and (**E–H**) *δ*D_CH4_ vs the relative amount of CH_4_ produced (*f*). Each data point is a sacrificed culture (see details in Materials and Methods). The symbols represent methane measured in the headspace of a single tube, and the gray shades represent replicates of the mutant strains. The initial measured *δ*^13^C and *δ*D of the methanol were −41.6‰ and −49‰, respectively, and the measured δD of the water was −79‰. The model-fitted *δ*^13^C_CH4_ and *δ*D_CH4_ values were obtained using the median values of the posterior KIEs as given in Table 2.

The δ values are reported relative to the VPDB (Vienna Pee Dee Belemnite) isotopic reference scale. *δ*^13^C_CH4_ values increased as methane production approached completion, that is, *f =* 1, where *f =* [CH_4_]_*t*_/[CH_4_]_final_, as expected for Rayleigh isotopic distillation in a closed system (Fig. 3A through D). The *δ*^13^C_CH4_ at the start of an experiment (i.e., when *f* is ~0) ranged between −111‰ and −103‰. These values, given the initial δ^13^C of the methanol (*δ*^13^C_CH3OH_ of −41.7‰), allow us to estimate the approximate value of ^13^ε_CH4-methanol_ to be between −72‰ and −64‰ (Table S7), which is within the range observed in previous studies (Fig. 1A). These data indicate that our growth conditions are comparable to prior experiments. The *δ*^13^C_CH4_ at the stationary phase, that is, when the methanol was fully consumed, was comparable between WT and the mutants, and the values ranged between −55‰ and −48‰. These values are lower than the initial *δ*^13^C_CH3OH_ (−41.7‰) and are within the range of differences observed in previous studies with *M. acetivorans* as well as with other methanogens grown on methanol (*δ*^13^C_CH4_ 8‰–12‰ more negative than the initial *δ*^13^C_CH3OH_) (9, 13, 25, 44). This difference indicates that the substrate is converted to multiple products that have different isotopic compositions (5).

Like *δ*^13^C_CH4_, the hydrogen isotopic composition of methane (*δ*D_CH4_) increased with methane production, where:


(3) $$\delta D_{CH4}={\left(\frac{\left[{}^{2}H\right]}{\left[{}^{1}H\right]}\right)}_{CH4}/{\left(\frac{\left[{}^{2}H\right]}{\left[{}^{1}H\right]}\right)}_{VSMOW}-1$$

The *δ*D values are reported relative to the VSMOW (Vienna Standard Mean Ocean Water) isotopic reference scale. At the stationary phase, *δ*D_CH4_ values were similar between WT and the mutants, with values ranging between −257‰ and −252‰ (Fig. 3E through H). The final *δ*D_CH4_ at the stationary phase is set by both the isotopic composition of the water in the growth medium (δD_H2O_), which, in our experiments, had a *δ*D of −79‰, and the initial *δ*D of the methyl group of the methanol, which had a *δ*D of −49‰ (see Materials and Methods). Consequently, and unlike the carbon isotope data, the *δ*D_CH4_ cannot be compared across different studies in a straightforward manner, as the *δ*D_H2O_ of the growth medium can vary by >100‰ between laboratory locations that span continents (45).

### A Bayesian approach to estimate kinetic isotope effects in a physiological context

We calculated enzymatic KIEs using the measured *δ*^13^C_CH4_ and *δ*D_CH4_ values (Fig. 3) in the context of a published reaction network of methylotrophic methanogenesis (9). This reaction network includes the activation of methanol catalyzed by MTA to generate CH_3_-S-CoM and its subsequent disproportionation to CH_4_ and CO_2_, as well as a biomass sink of 10% (Fig. 1B; Fig. S1). We assume a constant ratio of 3:1 between CH_4_ and CO_2_ over the course of a growth cycle. Based on a previously published metabolic model of the closely related strain *Methanosarcina barkeri*, we assume that the MTA- and MCR-catalyzed reactions are irreversible (especially under substrate-replete conditions used for our growth experiments) and that methanol diffusion is sufficiently rapid that it neither limits growth nor sets final methane isotopic compositions (41). We permit a partial reversibility of 0.05 in the oxidative branch based on a prior study where we focused on constraining degrees of reversibility of the pathway using the same growth conditions used here (9). We also examined how these choices affect our final conclusions via sensitivity testing (see below).

The model employed in this study can be described mathematically as a series of ordinary differential equations that quantify the rates of production and/or consumption of CH_3_OH, CO_2_, and CH_4_ (see Materials and Methods) and the respective mass balance for the isotopically unsubstituted and singly substituted species. To run the model, the initial *δ*^13^C and *δ*D values of the methanol and δD_H2O_ must be independently known (as is the case in our study). We assume that the concentration and isotopic compositions of the intermediates are at a steady state between integration steps, a common assumption of isotopic models (46). The output of the model includes the time-dependent concentrations for CH_3_OH, CO_2_, CH_4_, and their respective *δ*^13^C_CH3OH_, *δ*D_CH3OH_, *δ*^13^C_CO2_, *δ*^13^C_CH4_, and *δ*D_CH4_ values. As growth occurs in hermetically sealed tubes, we assume the system is closed such that methane accumulates in the system during growth and is well-mixed when sampled. We used our isotopically enabled metabolic model to calculate the enzyme-specific KIEs for MTA using a Markov Chain Monte Carlo (MCMC) algorithm to fit the data as previously described (9) (see model parameters in Table S4). The algorithm generates posterior distributions of the KIEs that generate the best fit to the experimentally measured *δ*^13^C_CH4_ and *δ*D_CH4_ values. Because KIEs are often small with values close to unity, we report them using the ε notation (equation 1) where *ε* = KIE − 1 (in ‰). We verified that our chosen bounds do not affect the best-fit values of the isotope effects.

In addition to the KIEs of MTA, we also calculated the ratio of the KIEs of MCR and the first enzyme in the oxidative branch, methyl-tetrahydromethanopterin:coenzyme M methyltransferase (MTR) (^13^KIE_MCR_/^13^KIE_MTR_ ≡ ^13^KIE_MCR/MTR_), which controls the *δ*^13^C_CH4_ values at the end of the experiment when *f* = 1. Although our metabolic framework, in principle, allows us to constrain the KIEs of all reactions in the methanogenic pathway, we chose not to extend our analysis beyond MTA and the ^13^KIE_MCR/MTR_ because measurement of *δ*^13^C_CH4_ and *δ*D_CH4_ values alone do not provide sufficient constraints to solve for additional free parameters downstream of these reactions. This lack of sensitivity is shown by the posterior distributions of these model parameters in Fig. S3 and S4.

### Large carbon kinetic isotopic effects associated with methanol-specific methyltransferases

Using the MCMC fitting algorithm outlined above, we calculated the carbon KIE of MTA (^13^*ε*_MTA_) for WT and the three mutants expressing a single copy of each of the three *mtaCB* operons. The median ^13^*ε*_MTA_ for WT was $-{\text{66.1}}_{-\text{7.7}}^{+\text{2.6}}\text{‰}$, where the uncertainty represents the lower and upper bounds of 95% of the model results (which need not be symmetric). The median ^13^*ε*_MTA_ of the mutants was $-{\text{67.9}}_{-\text{5.1}}^{+\text{3.2}}\text{‰}$ for MtaC1B1A1, $-{\text{62.0}}_{-\text{5.5}}^{+\text{3.5}}\text{‰}$ for MtaC2B2A1, and $-{\text{61.2}}_{-\text{6.3}}^{+\text{6.4}}\text{‰}$ for MtaC3B3A1 (Table 2; Fig. 3A through D). These values are statistically indistinguishable from each other and from WT (*P* value > 0.05, see Materials and Methods). We also used the MCMC fitting algorithm to analyze a previously published data set for methanol growth at 37°C for *M. acetivorans* (13), which yielded ^13^*ε*_MTA_ values of $-{\text{73.0}}_{-\text{15.1}}^{+\text{10.8}}‰$ and $-{\text{71.1}}_{-\text{5.1}}^{+\text{6.4}}‰$, based on separately fitting their measured *δ*^13^C_CH3OH_ and *δ*^13^C_CH4_ values, respectively (Table S5). Both values are within the uncertainty range of our estimated ^13^*ε*_MTA_ for the WT and the mutants.

**TABLE 2:** Calculated carbon and hydrogen KIEs for *M. acetivorans^a^*

MtaCB isozyme	Strain	^13^*ε*_MTA_ (‰)			^13^KIE_MCR/MTR_			^2^*ε*_MTA_ (‰)		
		−	Median	+	−	Median	+	−	Median	+
All	WWM1	7.7	−66.1	2.6	0.011	0.982	0.027	10	−56	9
C1B1	WWM176	5.1	−67.9	3.2	0.011	0.981	0.020	12	−55	10
C2B2	WWM170	5.5	−62.0	3.5	0.014	0.975	0.015	14	−56	11
C3B3	WWM184	6.3	−61.2	6.4	0.022	0.979	0.024	13	−57	10

^*a*^ The values for the mutant strains (WWM170, WWM176, and WWM184) represent pooled posterior distributions of three replicate experiments, and the uncertainties represent 95% of the model results. The data for each replicate are shown in Table S6. The values of ^13^*ε*_MTA_, ^2^*ε*_MTA_, and ^13^KIE_MCR/MTR_ were statistically indistinguishable between the strains (see Materials and Methods).

Unlike MTA, we cannot estimate the absolute values of the carbon KIEs of MCR and MTR. To do so, we would require additional experimental data, such as the isotopic composition of metabolic intermediates in the methanogenic pathway (e.g., CH_3_-S-CoM), which is not, to our knowledge, currently possible. However, the model does allow us to estimate the ratio of the KIEs of the two enzymes (^13^KIE_MCR/MTR_). The model yields ^13^KIE_MCR/MTR_ to be ${\text{0.982}}_{-\text{0.011}}^{\text{+0.027}}$ for the WT, which is indistinguishable from the mutant strains with ^13^KIE_MCR/MTR_ of ${\text{0.981}}_{-\text{0.011}}^{\text{+0.020}}$ for MtaC1B1A1, ${\text{0.975}}_{-\text{0.014}}^{\text{+0.015}}$ for MtaC2B2A1, and ${\text{0.979}}_{-\text{0.022}}^{\text{+0.024}}$ for MtaC3B3A1 (Table 2). If we use the previously measured value of ^13^*ε*_MCR_ ≈ −40‰ ± 10‰ from *Methanothermobacter marburgensis* (47), we obtain ^13^*ε*_MTR_ values between −15‰ to −22‰ for the highest and lowest ^13^KIE_MCR/MTR_ values of 0.975 and 0.982, respectively. Since *M. marburgensis* is a thermophilic methanogen that grows at 60°C, it is not currently known if the ^13^*ε*_MCR_ from this strain is representative of a distantly related, mesophilic methanogen like *M. acetivorans*. Constraining ^13^*ε*_MTR_ confidently requires an independent estimate of ^13^*ε*_MCR_ of the enzyme purified from *M. acetivorans* at 37°C, which is beyond the scope of this study.

We conducted a variety of sensitivity analyses to test whether the various model assumptions impact the results presented above. Specifically, we tested whether the size of the biomass sink and the degree of reversibility of the oxidative branch affect the ^13^*ε*_MTA_ and ^13^KIE_MCR/MTR_ values (Table S6). We found that the size of the biomass sink (between 5% and 15%) had a negligible impact on the calculated ^13^*ε*_MTA_ values (<1.8‰) and on the ^13^KIE_MCR/MTR_ values (<0.009) (Table S6). Specifically, these changes are within the 95% uncertainty range of our model results and would have no impact on the median values or the outcome of statistical tests. Similarly, changing the reversibility in the oxidative branch between 0 (i.e., irreversible) and 0.2 had a small effect on the ^13^*ε*_MTA_ (<1.9‰) and ^13^KIE_MCR/MTR_ values (<0.008), which were also within the uncertainty bounds of the model results. Relaxing the assumption that MTA is irreversible by increasing its reversibility to 0.2, changed the ^13^*ε*_MTA_ value by up to 4‰, which was also within the uncertainty bounds of our model (Table S6). We note that at higher degrees of reversibility, the uncertainty levels become too large to determine the KIEs.

### Small hydrogen kinetic isotopic effects associated with methanol-specific methyltransferases

We used our MCMC algorithm to calculate the hydrogen KIE of MTA (^2^*ε*_MTA_). This is a secondary isotope effect as the methyltransferase reaction does not break or create any new C-H bonds. Secondary hydrogen KIEs are typically smaller in absolute magnitude than primary isotope effects (48). The median ^2^*ε*_MTA_ was $-{\text{56}}_{-\text{10}}^{+\text{9}}\text{‰}$ for the WT strain, which is indistinguishable from values for all mutant strains: $-{\text{55}}_{-\text{12}}^{+\text{10}}\text{‰}$ for MtaC1B1A1, $-{\text{56}}_{-\text{14}}^{+\text{11}}\text{‰}$ for MtaC2B2A1, and $-{\text{57}}_{-\text{13}}^{+\text{10}}\text{‰}$ for MtaC3B3A1 (Table 2; Fig. 3E through H). To the best of our knowledge, these are the first estimates of ^2^*ε*_MTA_, so we cannot compare our data to previous studies.

Unlike carbon isotope effects, we cannot estimate the ratios of the hydrogen KIEs for MCR and MTR because this ratio depends on three parameters: the KIE of MTR and the primary and secondary KIEs of MCR. As shown in Fig. S4, the dependence on three KIEs does not allow for a unique solution of any of their ratios. Finally, we conducted a sensitivity test of our assumption that MTA is irreversible. Specifically, we calculated ^2^*ε*_MTA_ for MTA’s reversibility from 0 (irreversible) to 0.2 and found that increasing the reversibility of MTA lowers the KIE, that is, makes ^2^*ε*_MTA_ less negative, by up to 34‰ (Table S6). While this is a significant change, it does not impact our overall conclusion that ^2^*ε*_MTA_ is a normal isotope effect (i.e., KIE < 1) that is small in magnitude.

## DISCUSSION

Methylotrophic methanogens growing on methanol have long been known to express large carbon isotope effects (11). We find that the isozyme-specific ^13^*ε*_MTA_ values range between −61.2‰ and −67.9‰ and contribute to ≈90% of the overall carbon isotope effect relative to the source methanol (Table S7), with the rest coming from the branching point between MCR and the oxidative pathway. These results support the prior hypothesis that large isotopic effects in methylotrophic methanogenesis emerge due to the KIE of the substrate-specific methyltransferase reaction (4). By performing growth experiments and isotopic measurements on mutants expressing a single copy of *mtaCBA*, we can also conclude that all of the isozymes have similar KIEs (Table 2). We note that our analysis included only MtaA1 and not the MtaA2 isozyme, since the former is highly expressed and required for methanol growth (49). The *δ*^13^C_CH4_ values at the end of the growth experiments were consistently lower than the initial *δ*^13^C_CH3OH_ values by ≈9‰ (Table S2), and accordingly, we found that ^13^*ε*_MCR_ is consistently larger in absolute magnitude than ^13^*ε*_MTR_, with KIE ratios translating to a difference of ~15‰. However, this difference contributed to only ≈10% of the overall carbon isotopic offset between methane and methanol (Table S7).

Our experimentally measured carbon and hydrogen KIEs can be used to understand the biochemistry of the underlying enzymes and their reaction mechanisms (48, 50). The methylation of the MtaC-bound cob(I)amide catalyzed by MtaB is thought to proceed via an S_N_2 reaction mechanism, in which the carbon in the methanol is activated by Zn(II), a strong electrophile, in MtaB and then attacked by the strong nucleophile, a cob(I)amide in MtaC (see Fig. 3 in reference 51). S_N_2 methylation reactions commonly have small (in absolute terms) normal or inverse secondary hydrogen KIEs that range between −100‰ and +100‰ (52). This contrasts with S_N_1 mechanisms, which commonly have larger normal secondary hydrogen KIEs of up to −350‰ (52). In addition, S_N_2 reactions often have relatively large carbon KIEs that range between −40‰ and −80‰ (52–55). Thus, our findings of a small hydrogen KIE (≈−55‰) and large carbon KIE (≈−65‰) support an S_N_2 reaction mechanism for the MtaB-mediated methyltransferase reaction.

Our experimentally measured carbon and hydrogen KIEs also have implications for interpreting isotopic compositions of methane in natural environments. The impact of environmental conditions on the isotopic composition of methane has been clearly demonstrated for methanogenic growth on H_2_ and CO_2_ (hydrogenotrophic methanogenesis) (e.g., reference 8). Under high nutrient conditions in the laboratory, hydrogenotrophic methanogens generate methane with isotopic compositions that are similar to those detected in high-energy environments (such as near-surface terrestrial systems) and distinct from those detected in low-energy environments (like deep-sea sediments) (56). These differences have been attributed to changes in enzyme reversibility as a function of the amount of free energy (∆G) available in the environment to drive methanogenesis (7–9, 57). It has also been proposed that methylotrophic growth at lower ΔG gradients can lead to the expression of equilibrium hydrogen isotope effects driven by enzyme reversibility (58). It is unclear if this is also the case for carbon isotopes. The theoretically determined carbon isotopic equilibrium between CH_4_ and methanol is smaller than ^13^*ε*_CH4-methanol_ observed in laboratory cultures, though with the same sign (~−20‰ vs ~−70‰, respectively, reference 59). Isotopic equilibration between methanol and methane requires that MTA be fully reversible. *In vitro* data suggest that the reaction catalyzed by MtaB is reversible at high enzyme concentrations (60). However, environments that have low methanol concentrations between 1 and 10 μM and are dominated by methylotrophic methanogenesis produce *δ*^13^C_CH4_ values as low as ≈−90‰ vs a *δ*^13^C of ≈−20‰ for the total organic carbon, which translates to a ^13^*ε*_CH4-methanol_ of ≈−70‰, similar to values observed in substrate-replete conditions (27). Hence, it seems unlikely that MTA will generally be sufficiently reversible under environmental conditions to drive equilibration of either carbon or hydrogen isotopes in methane with methanol.

Importantly, it is possible to modify the isotopic composition of methane without altering the reversibility of MTA. This can be done by increasing the reversibility of the oxidative branch and MCR, which could result in hydrogen and carbon isotope exchange and even equilibration between methane and water for hydrogen isotopes, and methane and CO_2_ for carbon isotopes, regardless of the reversibility of the MTA-catalyzed reaction (8, 9). Reversibility in the branched portion of the network could occur given a sufficiently low environmental ΔG, for example, due to low substrate concentrations. We propose that such equilibration is more likely for hydrogen isotopes and less so for the carbon isotopes. This is because there are four different enzymes that exchange hydrogen isotopes with water, and just two of them would overwrite the KIEs of MTA if the reversibility was high enough. In contrast, exchange of carbon isotopes would require all the steps between CH_4_ and CO_2_ to be reversible (Fig. S1), which is possible but requires lower ΔG gradients (i.e., closer to 0) (7, 8). This decoupling between hydrogen and carbon isotopes has implications for interpreting *δ*D_CH4_ and *δ*^13^C_CH4_ values of methylotrophic methanogenesis in natural environments. Specifically, we predict that methylotrophic methanogenesis from methanol will have a large carbon isotope offset across a wide range of ΔG gradients, while the *δ*D_CH4_ values would trend towards isotopic equilibrium with the water as the ΔG available to the cells decreases (9). Such relationships could also be seen in measurements of multiple substituted (“clumped”) isotopologues of methane (^13^CH_3_D and ^12^CH_2_D_2_; references 61–64), which would also tend toward equilibrated values with increased degrees of reversibility in the oxidative branch. Incorporation of the KIEs modeled here for bulk isotopes in a full metabolic model of methylotrophic methanogenesis will be useful for the future interpretation of combined measurement of *δ*^13^C, *δ*D, and clumped isotopes of methane, as has been done for the hydrogenotrophic pathway (7, 26, 44, 59, 65–68).

Altogether, our experimentally measured KIEs support the hypothesis that large carbon KIEs of MTA dominate the large ^13^*ε*_CH4-methanol_ values observed in lab cultures, and that the branching point has a minor but measurable contribution to ^13^*ε*_CH4-methanol_. Our findings indicate that the KIEs of the three isozymes are indistinguishable, so while the relative levels of these three isozymes can change based on the growth phase, the overall isotopic signatures will remain similar across different environments, especially since MTA is predicted to have low reversibility across a wide range of methanol levels. This work provides support for the use of strongly ^13^C-depleted methane as an indicator of methylotrophic methanogenesis on methanol. Furthermore, the approach developed here can be expanded to the study of methanogenesis from compounds like methylamines and dimethyl sulfide to determine substrate-specific enzymatic KIEs and their role in the environment.

## MATERIALS AND METHODS

### Strains and growth media

The *Methanosarcina acetivorans* strains used in this study were described previously and are listed in Table 1. We used WWM1 (Δ*hpt*) (42) as the “wild-type” *M. acetivorans* strain for all growth experiments. The strains WWM170, WWM176, and WWM184 express a synthetic operon comprised of *mtaC2B2A1*, *mtaC1B1A1*, and *mtaC3B3A1*, respectively, in a Δ*mtaC1B1*Δ*mtaC2B2*Δ*mtaC3B3*Δ*mtaA1*Δ*mtaA2* deletion mutant and under a tetracycline-inducible *mcr* promoter (Δ*hpt*::P*mcrB-tetR*-𝜙C31intattP::P*mcrB*(*tetO1*)-*mtaCXBXA1*, where “*X*” stands for the 1, 2, or 3 isoforms [43]). These strains, in principle, should grow on methanol only if the growth medium is supplemented with tetracycline to induce expression of the *mtaCXBXA1* operon. However, our growth experiments indicated that WWM170, WWM176, and WWM184 can grow on methanol even in the absence of tetracycline (Fig. S5). In addition, there was no observable difference in the growth rates when the mutants were grown on 1, 10, and 100 μg/mL tetracycline (Fig. S2). Whole-genome sequencing of WWM170, WWM176, and WWM184 (Tables S8 to S10) revealed that each of them has a disruption in the *tetR* open reading frame, which leads to constitutive expression of the *mtaCXBXA1* operon. Thus, the different tetracycline treatments were regarded as biological replicates.

All strains were grown in single-cell morphology at 37°C without shaking (HeraTherm General Protocol Microbiological Incubator, Thermo Fisher Scientific) in PIPES-buffered HS liquid medium (69) with a headspace of helium at an overpressure of 70 kPa. The medium was supplemented with 125 mM methanol as the sole carbon source. Anaerobic, sterile fresh stocks of tetracycline hydrochloride were prepared in deionized water before use and added to final concentrations between 1 and 100 μg/mL^−1^. Growth was conducted in 26 mL Balch tubes with 10 mL medium.

### Cultivation for isotopic assays

All growth experiments were initiated with stationary phase cultures pre-grown in 50 mM trimethylamine·hydrochloride (TMA). The cells were transferred to HS medium with 125 mM methanol. Each experiment was initiated with five tubes for each replicate (three replicates for each mutant strain) or with a total of 10 tubes for the WT. Growth was monitored with optical density measurements at 600 nm (OD_600_), using a UV-Vis Spectrophotometer (Genesys 50, Thermo Fisher Scientific) with a blank containing 10 mL uninoculated HS media (Fig. S2). The experiments were concluded when the OD_600_ of the cultures did not change for at least 48 hours. Tubes that contained tetracycline were wrapped in tin foil to avoid light-dependent degradation of tetracycline over time. Growth was arrested along the growth curve by the injection of 1 mL of 1 M NaOH solution. Following NaOH injection, tubes were maintained at room temperature for downstream analysis of CH_4_ in the headspace (see Methane concentration determination and Measurement of carbon and hydrogen isotopic composition of methane) and whole-genome sequencing (see DNA extraction and sequencing). We note that cells were not washed before inoculation. While TMA and dimethylamine (DMA) are likely fully consumed when cells reach the stationary phase, some amount of monomethylamine (MMA) may remain and can be transferred along with the inoculum to medium with methanol used for growth and isotope ratio measurements (70). We estimate to have maximal MMA carryover levels of 0.5 mM, ≈250-fold lower than the initial methanol concentration in the growth medium, which we expect would have a negligible impact on the isotopic composition of the methane produced in our experiments.

### DNA extraction and sequencing

We performed whole-genome resequencing of the mutant strains used in this study to confirm the genotype reported by Opulencia et al. (43). We extracted the genomic DNA for whole-genome re-sequencing of strains WWM170, WWM176, and WWM184 from 10 mL of stationary phase culture, using the Qiagen blood and tissue kit (Qiagen, Hilden, Germany). Genomic DNA sequencing was performed by SeqCenter (Pittsburgh, PA), where our samples were further processed using the Illumina DNA Prep kit. We mapped the sequencing reads (150 bp paired-end reads) to a genome of the *M. acetivorans* strain WWM75 (71), using breseq v.0.35.5 (72). The complete list of the mutations that the breseq analysis identified is given in Tables S8 to S10.

### Methane concentration determinations

CH_4_ and He concentrations in the headspace were measured at the end of the experiment using a Clarus 590 Gas Chromatograph (GC) (Perkin-Elmer) outfitted with a HayeSep 1/8″ N60/80-6ft column (Perkin-Elmer) and a 100 μL sample loop, using Ar with a flow rate of 27 mL min^−1^ as a carrier gas. CH_4_ and He were detected with a flame ionization detector (FID) and thermal conductivity detector (TCD), respectively. A calibration curve was conducted with methane in known volumes prior to measurements. Relative changes in CH_4_ content in the headspace during a given growth experiment were tracked as the ratio of the peak areas of CH_4_ relative to He, which were used to calculate the methane product yield (*f*) (Table S2).

### Measurement of carbon and hydrogen isotopic composition of methane

The methane carbon and hydrogen isotopic compositions (*δ*^13^C and *δ*D) were determined at the Center for Isotope Geochemistry at the Lawrence Berkeley National Laboratory using a gas-chromatograph isotope ratio mass spectrometer (GC-IRMS) system (Thermo Scientific GC TraceGas Ultra system connected to a Thermo Scientific Delta V Plus IRMS), as described previously (56). Briefly, the gases were sampled directly from the Balch tube headspace using a gas-tight syringe and injected into a stainless-steel loop with a chosen size based on sample concentration (5–100 μL) attached to a 6-port valve (VICI-Valco). The methane was separated chromatographically on an HP-mole sieve fused silica capillary column (30 m × 0.32 mm) using He as carrier gas. Following chromatographic separation, CH_4_ was passed through a ceramic tube at 1,200°C, combusted, and converted to CO_2_ for carbon isotopic measurements, or through a ceramic tube at 1,420°C, pyrolyzed, and converted to H_2_ for hydrogen isotopic measurements. For hydrogen isotopic measurements, ceramic tubes were pre-conditioned by injecting 250 μL of pure methane three times the day before the measurement session. Measured *δ*^13^C and *δ*D values were corrected for instrument linearity and external natural gas standards obtained from the United States Geological Service HCG-1, HCG-2, and HCG-3 (73) (Table S2). The *δ*^13^C and *δ*D values of the methanol stocks were the same as those used in Gropp et al. (9) and measured as part of that study following methods in Lloyd et al. (74). Briefly, pure methanol was derivatized to CH_3_I using HI acid and then converted to chloromethane (CH_3_Cl) using excess silver chloride (AgCl). The isotopic composition of CH_3_Cl was measured using a MAT 253 Ultra (Thermo Scientific) at UC Berkeley and yielded a δD value of −49.0 ± 0.2‰ (1SE) and a *δ*^13^C value of −41.67 ± 0.01‰ (1SE) on the VSMOW scale and Vienna Peedee Belemnite scale (VPDB), respectively. The δD of the water (δD_H2O_) was measured for representative samples at the Center for Stable Isotope Biogeochemistry at UC Berkeley, via conversion of water to H_2_ using a hot chromium reactor unit (Thermo H/Device) connected to a Thermo Delta V Plus isotope ratio mass spectrometer. External water standards of known isotopic composition GFLES-2 (+159.9‰), GFLES-3 (+280.2‰), and GFLES-4 (+399.8‰) (USGS Reston) were used to correct samples to the VSMOW scale.

### *In silico* isotope-enabled metabolic modeling of methylotrophic methanogenesis

To calculate the enzyme-specific KIEs, we used a reaction network of the methylotrophic pathway during growth on methanol that includes the minimal set of reactions needed to model the measured isotope effects that we described in the Results section (Fig. S1). The model includes a biomass sink from methyl-tetrahydromethanopterin (CH_3_-H_4_MPT), which we set at 10% of the total flux of methanol into the pathway based on prior work (12). Together with the 3:1 ratio of the products CH_4_:CO_2_, this sink determines the disproportionation ratio of CH_3_-S-CoM between MCR and MTR. As mentioned in the Results section, we allowed partial reversibility in the oxidative branch following previously reported values of ~0.05 net reversibility (9). The reversibility of the *i*th reaction “$r_{i}$” is defined here as the ratio of the backward to forward fluxes of the reaction ($J_{i}^{-}$ and $J_{i}^{+}$, respectively), where $r_{i}=J_{i}^{-}/J_{i}^{+}$. We note that this value represents the net reversibility for hydrogen, which can be calculated as the average of the reversibility of each step in the pathway: $r_{net}=\left(\frac{1}{3}\right)\left(r_{3}\cdot r_{4}\cdot r_{5}+r_{3}\cdot r_{4}\cdot r_{5}+r_{3}\cdot r_{4}\right)$, where the numbers correspond to the reactions in Fig. S1. Since we assumed here that the reaction catalyzed by methylene–tetrahydromethanopterin dehydrogenase (MTD) is irreversible, that is, $r_{5}=0$, MTR and methylenetetrahydromethanopterin:coenzyme-F_420_ reductase (MER) were assigned, with a reversibility of 0.4 to obtain a net reversibility of 0.05. We provide the results of a sensitivity analysis in regard to the degree of reversibility in Table S6, and the isotopic model parameters in Table S4.

### Calculation of kinetic isotopic effects

We estimated ^13^ε and ^2^ε values using the MCMC algorithm implemented in the *emcee* package in Python (75). To determine which of these parameters could be resolved with the MCMC algorithm given the experimental results, we generated a synthetic data set with the model described in the “*In silico* isotope enabled metabolic modeling of methylotrophic methanogenesis” section. We found that the model reproduces the ^13^ε_MTA_ and ^2^ε_MTA_ and the ratios of the carbon KIEs of MCR and MTR (^13^KIE_MCR/MTR_) of the synthetic data set (Fig. S3 and S4). The algorithm works as follows: Using predefined boundaries for the KIEs and the initial δ^13^C_CH3OH_, δD_CH3OH_, and δD_H2O_ values, the MCMC algorithm solves the isotopic model multiple times while minimizing the distance from the modeled vs measured δ^13^C_CH4_ and δD_CH4_ values. The MCMC parameters in our simulations were 100 walkers, 600 steps, and 400 burn-in discarded steps, resulting in a total of 20,000 effective samples. We defined the allowable boundaries for the KIEs, based on typical measured ranges in the literature (48). For carbon KIEs, we bound ^13^ε to be between −100‰ and 0‰ (0.9 < KIE < 1). For hydrogen KIEs of reactions that break or create a bond with the hydrogen atom (primary KIEs), we used a ^2^ε range between −900‰ and 0‰ (0.1 < KIE < 1). For hydrogen KIEs of reactions that do not break or create a bond with the hydrogen atom (secondary KIEs), we used a smaller range of ^2^ε between −300‰ and +200‰ (0.7 < KIE < 1.2). For these secondary isotope effects, we allow for the possibility of inverse effects in which deuterated species react faster than isotopically unsubstituted species.

The posterior distributions of the KIEs were extracted from the MCMC algorithm and were used to estimate the uncertainty of the KIEs, which were defined by the 0.025 and 0.975 percentiles (95% of the posterior model results). In experiments involving mutant strains WWM170, WWM176, and WWM184, three replicates were conducted. For these, we pooled the posterior distributions to estimate uncertainties. Statistical significance was assessed by calculating the element-wise difference between parameter posterior distributions. A difference was considered significant if the 95% credible interval of the difference distribution excluded zero. When estimating the KIEs using MCMC, we incorporate the uncertainty in the measured product yield (*f*) and in the isotopic values by introducing noise that is proportional to the standard deviation of those parameters. Specifically, we use a relative uncertainty of 2% (1SD) in our *f* values, 0.07‰ in our δ^13^C_CH4_ values (1SD), and 2‰ for our δD_CH4_ values (1SD). In our comparisons of the synthetic data sets and previously published work, we use similar uncertainties to be internally consistent.

## Data Availability

All data are available in the article or the supplemental material. The DNA sequences of the mutant strains were deposited in the NCBI database at https://www.ncbi.nlm.nih.gov/bioproject/1444202. The code used in this manuscript was archived in Zenodo (DOI: 10.5281/zenodo.20617536).

## SUPPLEMENTAL MATERIAL

Supplemental material aem.00664-26-s0001.docx Supplemental text; Fig. S1 to S5.

Supplemental tables aem.00664-26-s0002.xlsx Tables S1 to S10.

ASM does not own the copyrights to Supplemental Material that may be linked to, or accessed through, an article. The authors have granted ASM a non-exclusive, world-wide license to publish the Supplemental Material files. Please contact the corresponding author directly for reuse.
