# Supplementary material for: Methanol-specific methyltransferase isozymes have large carbon kinetic isotope effects that impact methane isotopic signatures
Source: Appl Environ Microbiol. 2026 Jul 21;92(8):e00664-26. doi: 10.1128/aem.00664-26 (PMC13488299; doi:10.1128/aem.00664-26)
Supplement: Supplementary file 1 [file aem.00664-26-s0001.docx]

**Supplementary Materials for:**

**Methanol-specific methyltransferase isozymes have large carbon kinetic isotope effects that impact methane isotopic signatures**

Jonathan Gropp^1,2^, Markus Bill^3^, Daniel A. Stolper^1*^, Dipti D. Nayak^2,4*^

^1^Department of Earth and Planetary Science, University of California, Berkeley; Berkeley, CA, USA

^2^Department of Molecular and Cell Biology, University of California, Berkeley; Berkeley, CA, USA

^3^Energy Geosciences Division, Lawrence Berkeley National Laboratory; Berkeley, CA, USA

^4^Department of Plant and Microbial Biology, University of California, Berkeley; Berkeley, CA, USA

* Corresponding authors

*Corresponding authors:

Daniel A. Stolper (dstolper@berkeley.edu), Department of Earth and Planetary Science, University of California, Berkeley; Berkeley, CA 94720-3204.

Dipti D. Nayak (dnayak@berkeley.edu), Department of Molecular and Cell Biology, University of California, Berkeley, CA 94720-3204.

**Supplementary text**

**An isotopic mass balance of methylotrophic methanogenesis on methanol**

The following equations were used to track the time derivatives of the [CH_3_OH], [CH_4_], and [CO_2_], and their ^13^C/^12^C and D/H ratios. The reactions’ numbers correspond to the reactions’ network as presented in Fig. S1.

| $\frac{d\left[ CH_{3}OH \right]}{dt}=-k\left[ CH_{3}OH \right]=J_{1}=J_{1}^{+}-J_{1}^{-}=J_{1}^{+}\left( 1-r_{1} \right)$ | Eq. s1 |
| --- | --- |
| $\begin{aligned} \frac{d\left[ CH_{4} \right]}{dt}=\frac{d\left[ CH_{3}OH \right]}{dt}n\left( 1-Y_{b} \right)=J_{2} \end{aligned}$ | Eq. s2 |
| $\begin{aligned} \frac{d\left[ CO_{2} \right]}{dt}=\frac{d\left[ CH_{3}OH \right]}{dt}\left( 1-n \right)\left( 1-Y_{b} \right)=J_{4}=J_{5} \end{aligned}$ | Eq. s3 |
| $\begin{aligned} \frac{d\left[ biomass \right]}{dt}=\frac{d\left[ CH_{3}OH \right]}{dt}Y_{b}=J_{biomass}^{+} \end{aligned}$ | Eq. s4 |
| $\begin{aligned} J_{2}^{+}=J_{1}n(1-Y_{b})\left( 1-r_{2} \right) \end{aligned}$ | Eq. s5 |
| $\begin{aligned} J_{3}^{+}=J_{1}\left[ \left( 1-n \right)\left( 1-Y_{b} \right)+Y_{b} \right]\left( 1-r_{3} \right) \end{aligned}$ | Eq. s6 |
| $\begin{aligned} J_{4}^{+}=J_{1}\left( 1-n \right)\left( 1-Y_{b} \right)\left( 1-r_{4} \right) \end{aligned}$ | Eq. s7 |
| $\begin{aligned} J_{5}^{+}=J_{1}\left( 1-n \right)\left( 1-Y_{b} \right) \end{aligned}$ | Eq. s8 |
| $\begin{aligned} J_{biomass}^{+}=J_{1}Y_{b} \end{aligned}$ | Eq. s9 |

where $J_{i}$ is the metabolic flux of the *i*th reaction, and $J_{i}^{+}$ and $J_{i}^{-}$ denote its forward and reverse fluxes, respectively, $Y_{b}$ is the biomass sink, and *n* is the fraction of CH_3_-S-CoM molecules reduced to CH_4_ relative to CO_2_. The reversibility of the *i*th reaction $r_{i}$ is defined by $r_{i}=J_{i}^{-}/J_{i}^{+}$. In this work we used *n* = 0.75 which represents a CH_4_:CO_2_ ratio of 3:1, and $Y_{b}$ = 0.1 (1).

| $\begin{aligned} \frac{d{}^{13}{R_{CH_{3}OH}}}{dt}=\frac{J_{1}^{+}}{\left[ CH_{3}OH \right]}\left( r_{1}{}^{13}{R_{CH_{3}SCoM}}{{}^{13}\alpha}_{MTA}^{-}-{}^{13}{R_{CH_{3}OH}}\left( {}^{13}{\alpha_{MTA}^{+}}-1+r_{1} \right) \right) \end{aligned}$ | Eq. s10 |
| --- | --- |
| $\begin{aligned} \frac{d{}^{13}{R_{CH_{4}}}}{dt}=\frac{J_{2}^{+}}{\left[ CH_{4} \right]}\left( {}^{13}{R_{CH_{3}SCoM}}{{}^{13}\alpha}_{MCR}^{+}-{}^{13}{R_{CH_{4}}}\left( r_{2}{{}^{13}\alpha}_{MCR}^{-}+\left( 1-r_{2} \right) \right) \right) \end{aligned}$ | Eq. s11 |
| $\begin{aligned} \frac{d{}^{13}{R_{CO_{2}}}}{dt}=\frac{J_{4}^{+}}{\left[ CO_{2} \right]}\left( {}^{13}{R_{CH_{2}H_{4}MPT}}{{}^{13}\alpha}_{MTD}^{+}-{}^{13}{R_{CO_{2}}} \right) \end{aligned}$ | Eq. s12 |
| $\begin{aligned} \frac{d{}^{13}{R_{biomass}}}{dt}=\frac{J_{biomass}^{+}}{\left[ biomass \right]}\left( {}^{13}{R_{CH_{3}H_{4}MPT}}{{}^{13}\alpha}_{biomass}^{+}-{}^{13}{R_{biomass}} \right) \end{aligned}$ | Eq. s13 |

Here ${}^{13}{R_{j}}$ is the ^13^C/^12^C ratio of the *j*th metabolite, and ${{}^{13}\alpha}_{i}^{+}$ and ${{}^{13}\alpha}_{i}^{-}$ are the forward and reverse carbon KIEs of the i*th* reaction, respectively.

We assumed that the intermediate metabolites are at a steady state between the integration steps so that:

| ${}^{13}{R_{CH_{3}SCoM}}=\frac{J_{1}^{+}{}^{13}{R_{CH_{3}OH}}{}^{13}{\alpha_{MTA}^{+}}+J_{2}^{+}r_{2}{}^{13}{R_{CH_{4}}}{}^{13}{\alpha_{MCR}^{-}}}{w\left( 1-\frac{j}{w}J_{3}^{+}r_{3}{}^{13}{\alpha_{MTR}^{-}} \right)}$ | Eq. s14 |
| --- | --- |

where:

| $\begin{aligned} j=\frac{J_{3}^{+}{}^{13}{\alpha_{MTR}^{+}}}{\begin{aligned} \left( 1-\frac{\left( \left( J_{4}^{+} \right)^{2}r_{4}{}^{13}{\alpha_{MER}^{-}}{}^{13}{\alpha_{MER}^{+}} \right)/\left( J_{4}^{+}r_{4}{}^{13}{\alpha_{MER}^{-}}+J_{5}^{+}{}^{13}{\alpha_{MTD}^{+}} \right)}{J_{3}^{+}r_{3}{}^{13}{\alpha_{MTR}^{-}+}J_{4}^{+}{}^{13}{\alpha_{MER}^{+}}+J_{biomass}^{+}{}^{13}{\alpha_{biomass}^{+}}} \right)\times\\ \left( J_{3}^{+}r_{3}{}^{13}{\alpha_{MTR}^{-}+}J_{4}^{+}{}^{13}{\alpha_{MER}^{+}}+J_{biomass}^{+}{}^{13}{\alpha_{biomass}^{+}} \right) \end{aligned}}\# \end{aligned}$ | Eq. s15 |
| --- | --- |
| $\begin{aligned} w=J_{1}^{+}r_{1}{}^{13}{\alpha_{MTA}^{-}}+J_{2}^{+}{}^{13}{\alpha_{MCR}^{+}}+J_{3}^{+}{}^{13}{\alpha_{MTR}^{+}} \end{aligned}$ | Eq. s16 |
| $\begin{aligned} {}^{13}{R_{CH_{3}H_{4}MPT}}={}^{13}{R_{CH_{3}SCoM}}\times j \end{aligned}$ | Eq. s17 |
| $\begin{aligned} {}^{13}{R_{CH_{2}H_{4}MPT}}=\frac{J_{4}^{+}{}^{13}{R_{CH_{3}H_{4}MPT}}{}^{13}{\alpha_{MER}^{+}}}{J_{4}^{+}r_{4}{}^{13}{\alpha_{MER}^{-}+J_{5}^{+}{}^{13}{\alpha_{MTD}^{+}}}} \end{aligned}$ | Eq. s18 |

We constructed a similar mass balance for hydrogen isotopes. Here the KIEs are denoted by the ‘s’ and ‘p’ superscripts that represent secondary and primary hydrogen KIEs, respectively.

| $\frac{d{}^{2}{R_{CH_{3}OH}}}{dt}=\frac{J_{1}^{+}}{\left[ CH_{3}OH \right]}\left( r_{1}{{}^{2}R}_{CH_{3}SCoM}{}^{2}{\alpha_{MTA}^{s-}}-{{}^{2}R}_{CH_{3}OH}\left( {}^{2}{\alpha_{MTA}^{s+}-1}+r_{1} \right) \right)$ | Eq. s19 |
| --- | --- |
| $\frac{d{}^{2}{R_{CH_{4}}}}{dt}=\frac{J_{2}^{+}}{4\left[ CH_{4} \right]}\left( \begin{aligned} {3{}^{2}R}_{CH_{3}SCoM}{}^{2}{\alpha_{MCR}^{s+}}+{3{}^{2}R}_{H_{2}O}{}^{2}{\alpha_{MCR}^{p+}}{}^{2}{\alpha_{H_{2}O,CoB}^{eq}}- \\ {{}^{2}R}_{CH_{4}}\left( r_{2}3{}^{2}{\alpha_{MCR}^{s-}}+r_{2}{}^{2}{\alpha_{MCR}^{p-}}-4\left( 1-r_{2} \right) \right) \end{aligned} \right)$ | Eq. s20 |
| $\frac{d{}^{2}{R_{biomass}}}{dt}=\frac{J_{2}^{+}}{\left[ biomass \right]}\left( {{}^{2}R}_{CH_{3}H_{4}MPT}{}^{2}{\alpha_{biomass}^{s+}}+{}^{2}{R_{biomass}} \right)$ | Eq. s21 |

Similarly to the carbon isotope mass balance, we assume that the intermediate metabolites are at a steady state between the integration steps so that:

| ${}^{2}{R_{CH_{3}SCoM}}=\frac{\begin{aligned} 3J_{1}^{+}{}^{2}{R_{CH_{3}OH}}{}^{2}{\alpha_{MTA}^{s+}}+3J_{2}^{+}r_{2}{}^{2}{R_{CH_{4}}}{}^{2}{\alpha_{MCR}^{s-}}+ \\ \left[ \frac{3J_{3}^{+}r_{3}{}^{2}{\alpha_{MTR}^{s-}}J_{4}^{+}r_{4}{}^{2}{R_{H_{2}O}}{}^{2}{\alpha_{H_{2}O,F420}^{eq}}{}^{2}{\alpha_{MER}^{p-}}}{\left( 1-j \right)\left( \begin{aligned} 3J_{3}^{+}r_{3}{}^{2}{\alpha_{MTR}^{s-}}+3J_{biomass}^{+}{}^{2}{\alpha_{biomass}^{s+}}+ \\ J_{4}^{+}\left( 2{}^{2}{\alpha_{MER}^{s+}}+{}^{2}{\alpha_{MER}^{p+}} \right) \end{aligned} \right)} \right] \end{aligned}}{\left( 1-3wJ_{3}^{+}r_{3}{}^{2}{\alpha_{MTR}^{s-}} \right)3\left( J_{1}^{+}r_{1}{}^{2}{\alpha_{MTA}^{s-}}+J_{2}^{+}{}^{2}{\alpha_{MCR}^{s+}}+J_{3}^{+}{}^{2}{\alpha_{MTR}^{s+}} \right)}$ | Eq. s22 |
| --- | --- |

where:

| $\begin{aligned} j=\frac{\left( \frac{4\left( J_{4}^{+} \right)^{2}r_{4}{}^{2}{\alpha_{MER}^{s+}}{}^{2}{\alpha_{MER}^{s-}}}{2J_{4}^{+}r_{4}{}^{2}{\alpha_{MER}^{s-}}+J_{5}^{+}\left( {}^{2}{\alpha_{MTD}^{s+}}+{}^{2}{\alpha_{MTD}^{p+}} \right)} \right)}{3J_{3}^{+}r_{3}{}^{2}{\alpha_{MTR}^{s-}}+3J_{biomass}{}^{2}{\alpha_{biomass}^{s+}}+J_{4}^{+}\left( 2{}^{2}{\alpha_{MER}^{s+}}+{}^{2}{\alpha_{MER}^{p+}} \right)} \end{aligned}$ | Eq. s23 |
| --- | --- |
| $\begin{aligned} w=\frac{\left( \frac{3J_{3}^{+}{}^{2}{\alpha_{MTR}^{s+}}}{\left( 1-j \right)\left( 3J_{3}^{+}r_{3}{}^{2}{\alpha_{MTR}^{s-}}+3J_{biomass}{}^{2}{\alpha_{biomass}^{s+}}+J_{4}^{+}\left( 2{}^{2}{\alpha_{MER}^{s+}}+{}^{2}{\alpha_{MER}^{p+}} \right) \right)} \right)}{3\left( J_{1}^{+}r_{1}{}^{2}{\alpha_{MTA}^{s-}}+J_{2}^{+}{}^{2}{\alpha_{MCR}^{s+}}+J_{3}^{+}{}^{2}{\alpha_{MTR}^{s+}} \right)} \end{aligned}$ | Eq. s24 |

**References**

1.	Londry KL, Dawson KG, Grover HD, Summons RE, Bradley AS. 2008. Stable carbon isotope fractionation between substrates and products of Methanosarcina barkeri. Organic Geochemistry 39:608–621.

**Supplementary Figures**

Fig. S1. **Metabolic network for the hydrogen and carbon isotopic mass balance.** The numbers and notations for the KIEs correspond to those used in Eqs. S1–S23. The green boxes represent the relative flux of carbon in the pathway. Reactions that break a C-H bond have both secondary and primary KIEs, denoted by ‘s’ and ‘p’ next to the α, respectively. The model assumes a 3:1 ratio of CH_4_:CO_2_ production, a biomass sink that is 10% of the total flux of methanol, and partial reversibility in the oxidative branch in the reactions catalyzed by MTR and Mer. The reactions catalyzed by MCH, FTR, and FMD do not affect the isotopic composition in methane because we assume that MTD is irreversible and thus they do not control the isotopic composition of the final methane. Abbreviations for enzyme complexes names and metabolites: CH_3_-S-CoM – methyl coenzyme M; H_4_MPT – tetrahydromethanopterin; MTA – methanol specific methyltransferase; MCR – methyl-CoM reductase; MTR – N^5^-methyl-H_4_MPT:coenzyme M methyltransferase; Mer – N^5^,N^10^-methylene-H_4_MPT:coenzyme-F420 reductase; MTD – N^5^,N^10^-methylene-H_4_MPT:coenzyme-F420 reductase. MCH – methenyl-H_4_MPT cyclohydrolase; FTR – formylmethanofuran:H_4_MPT formyltransferase; FMD – formylmethanofuran dehydrogenase.

fig. S2. **Growth curves of WT and mutant strains.** Optical density (OD) measurements were taken at 600 nm. The different symbols represent the replicates, where each symbol represents a single tube, with a total of 10 tubes for the WT and 5 per each mutant strain on a given tetracycline (tet) concentration. Tubes were sacrificed during the experiment by injection of 1 mL NaOH 1M for headspace analysis.

Fig. S3. **Test of carbon KIEs fits using the MCMC algorithm with synthetic data.** Here, the KIEs are denoted as ‘α’.The predefined KIEs were ${}^{13}{\alpha_{MTA}}$ = 0.935, ${}^{13}{\alpha_{MCR}}$ = 0.960, ${}^{13}{\alpha_{MTR}}$ = 0.980 (i.e., ${}^{13}{\alpha_{MCR}}$/${}^{13}{\alpha_{MTR}}$ = 0.980), and ${}^{13}{\alpha_{biomass}}$ of 0.98. The posterior distributions of ${}^{13}{\alpha_{MTA}}$ (${0.935}_{-0.006}^{+0.002}$) match the predefined value, while ${}^{13}{\alpha_{MCR}}$ and ${}^{13}{\alpha_{MTR}}$ do not match the predefined values and yield much larger uncertainties. However, the ratios of ${}^{13}{\alpha_{MCR}}$ and ${}^{13}{\alpha_{MTR}}$ yield a value of ${0.979}_{-0.013}^{+0.020}$, which matches the predefined value of 0.980. Analysis was performed using 250 walkers for 400 steps. The first 200 steps were discarded as burn-in, resulting in 50,000 effective samples used for parameter estimation. The diagonal histograms show the one-dimensional normalized posterior distributions for each parameter. The off-diagonal panels display the two-dimensional posterior distributions for all pairs of parameters, illustrating their correlations. Contours represent 68% (dark blue) and 95% (light blue) credible regions.

Fig. S4. **Test of hydrogen KIEs using the MCMC algorithm with synthetic data.** Here, the KIEs are denoted as ‘α’.The predefined KIEs were ${}^{2}{\alpha_{MTA}^{s}}$ = 0.950, ${}^{2}{\alpha_{MCR}^{s}}$ = 0.800, ${}^{2}{\alpha_{MCR}^{p}}$ = 0.700, and ${}^{2}{\alpha_{MTR}^{s}}$ = 0.9500. Analysis was performed using 10 walkers for 10,000 steps. The first 200 steps were discarded as burn-in, resulting in 98,000 effective samples used for parameter estimation. The posterior distributions of ${}^{2}{\alpha_{MTA}}$ (${0.950}_{-0.01}^{+0.01}$) match the predefined value. The three additional KIEs yield posterior distribution with large error estimates, and values that do not match the predefined values. The diagonal histograms show the one-dimensional normalized posterior distributions for each parameter. The off-diagonal panels display the two-dimensional posterior distributions for all pairs of parameters, illustrating their correlations. Contours represent 68% (dark blue) and 95% (light blue) credible regions. Note that the scales are different between the parameters, representing the prescribed bounds of each parameter.

Fig. S5. **Mutant strains grow exponentially without tetracycline addition.** Optical density (OD) measurements were taken at 600 nm. Strains were thawed from freezer stocks to HS-TMA media (50 mM) and then acclimated for growth on HS media with 60 mM methanol for one generation. Then 0.2 mL of late exponential phase culture was transferred in triplicates to HS media with 60 mM methanol but no tetracycline (replicates represented by the symbols).

**List of Supplementary Tables**

Table S1. Data compilation from previous work used to plot Fig. 1A.

Table S2. Growth data, carbon and hydrogen isotopic compositions of methane, and product yields.

Table S3. Growth parameters of the different strains used in this study.

Table S4. Isotopic parameters for the model.

Table S2. Fitting δ^13^C_CH3OH_ and δ^13^C_CH4_ from Penger et al., 2012.

Table S6. Sensitivity analysis for reversibility and the size of the biomass sink.

Table S7. The contribution of the KIE of MTA to the overall isotopic offset.

Table S8. Whole genome re-sequencing analysis with Breseq for WWM170.

Table S9. Whole genome re-sequencing analysis with Breseq for WWM176.

Table S10. Whole genome re-sequencing analysis with Breseq for WWM184.
